# CRISPR–Cas systems for plant virus management: detection, surveillance, and host resistance

**DOI:** 10.3389/fpls.2026.1804262

**Published:** 2026-07-02

**Authors:** Surender Kumar, Rahul Mohan Singh, Kiran R. Gadhave

**Affiliations:** 1 Texas A&M AgriLife High Plains Research and Extension Center, Canyon, TX, United States; 2 Department of Entomology, Texas A&M University, College Station, TX, United States

**Keywords:** CRISPR based diagnostics, field-based virus detection tool, plant virus surveillance, rapid virus detection, susceptibility gene editing

## Abstract

The CRISPR–Cas system has transformed genome manipulation by enabling precise and programmable modification of genetic material. Initially developed as a genome-editing tool, CRISPR technologies have expanded from fundamental research to applied use across plant, animal, and microbial systems due to their simplicity, accuracy, and versatility. In agriculture, CRISPR–Cas9 has progressed from crop improvement to host-directed strategies conferring resistance against a broad range of plant viruses. Concurrently, the discovery of additional Cas effector proteins, particularly Cas12a and Cas13a, has enabled highly sensitive nucleic acid–based diagnostic platforms supporting rapid, field-deployable pathogen detection. Here, we present a focused synthesis integrating CRISPR-mediated host resistance engineering with CRISPR-based diagnostic surveillance within a unified framework for plant virus management. Unlike previous reviews that treat these domains independently, we emphasize their convergence in enabling early detection, real-time surveillance, and targeted intervention across the disease cycle. Cas12a-based systems, currently the most widely implemented, have been coupled with isothermal amplification and visual readouts for rapid virus detection, whereas Cas13a-based platforms offer direct RNA targeting with potential for simplified workflows, although they remain less developed. We examine key design considerations, performance characteristics, and limitations of these platforms, including challenges related to sensitivity, multiplexing, and field deployment. Finally, we highlight future directions, including vector-based detection, multiplex diagnostics, and integration of CRISPR technologies into scalable surveillance systems. Collectively, this review positions CRISPR-based genome editing and diagnostics as complementary components of a next-generation strategy for plant virus detection, surveillance, and management.

## 1 Introduction

Food security is a central challenge of the twenty-first century; however, current agricultural productivity has reached a period of relative stagnation in the post–Green Revolution era. Crop yields are no longer increasing at sufficient rates to meet projected global food demands in the coming decades (Tilman et al., 2011; Ray et al., 2012; Bailey-Serres et al., 2019). Addressing this gap will require transformative interventions across multiple critical stages of agriculture, including yield enhancement, stress tolerance, and disease management. Innovative biotechnological approaches capable of overcoming these constraints are therefore essential for achieving sustainable agricultural growth (Noack et al., 2024; Hamdan et al., 2022; Karunarathna et al., 2021).

Plant pandemics are caused by a diverse set of cosmopolitan fungal, bacterial, and viral pathogens and continue to result in substantial yield losses worldwide (Ristaino et al., 2021; Wang et al., 2024). Notably, nearly 47% of documented plant disease pandemics are attributed to plant viruses, accounting for annual global losses estimated at approximately US$30 billion (Jones, 2021). Viral pandemics affect major staple crops, including maize lethal necrosis disease caused by mixed infections involving maize chlorotic mottle virus and one or more of sugarcane mosaic virus, maize dwarf virus, or wheat streak mosaic virus (Tatineni and Hein, 2023; Ristaino et al., 2021). Sterility mosaic disease caused by two related yet distinct emaraviruses named pigeonpea sterility mosaic virus-I and -II caused annual loss of approximately US$300 million loss and moreover if infection occurs in early stages of growth (<45 days), it can cause 100% crop loss (Reddy et al., 1998; Kumar et al., 2017). Tomato spotted wilt virus (TSWV) causes annual economic losses exceeding US$1 billion across multiple specialty and staple food crops in the United States (Mandal et al., 2001; Sevik and Arli-Sokmen, 2012). In tuber crops, viruses such as potato virus Y, potato leafroll virus, potato virus X, cassava mosaic virus, sweet potato mild mottle virus, and sweet potato chlorotic stunt virus impose severe yield penalties across sub-Saharan Africa, Southeast Asia, and the Indian subcontinent (Jones, 2021). Viral diseases also cause widespread losses in legumes, horticultural, and floricultural crops, including faba bean necrotic yellows disease, tomato yellow leaf curl disease, tomato brown rugose fruit disease, and viral diseases of pome, stone, and citrus fruits (Kumari and Makkouk, 2007; Salem et al., 2016; Yan et al., 2025; Kumar et al., 2012; Bar-Joseph, 2025).

Effective disease management relies on diagnostic tools that are cost-effective, rapid, reliable, and suitable for point-of-care testing without the need for sophisticated instrumentation (van Dongen et al., 2020). Traditionally, pathogen detection has depended on biological characterization, serological methods such as enzyme-linked immunosorbent assay (ELISA), and nucleic acid–based techniques including polymerase chain reaction (PCR) and reverse transcription-PCR (RT-PCR) (Henson and French, 1993; Clark and Adams, 1977; Kumar et al., 2012, Kumar et al., 2013). While these methods are highly sensitive, they require expensive reagents, specialized equipment, controlled laboratory conditions, longer detection time and trained personnel. Therefore, these tools remain limited to labs. Advances in isothermal amplification techniques such as loop-mediated isothermal amplification (LAMP) and recombinase polymerase amplification (RPA), along with serological approaches like lateral flow immunoassays (LIFA), have enabled the development of field-deployable, farmer-friendly diagnostic assays (Zhao et al., 2015; Bhat et al., 2022). Nevertheless, these tools remain limited in pathogen coverage and are susceptible to false positives and inhibition by compounds present in plant tissues (Aglietti et al., 2024).

RNA silencing (RNAi) represents a fundamental innate defense mechanism in plants against viral infection. However, plant viruses have evolved countermeasures by encoding RNA silencing suppressor (RSS) proteins that interfere with key components of the RNAi machinery (Ding and Voinnet, 2007; Díaz-Pendón and Ding, 2008). To mitigate viral impacts, resistant cultivars have been developed through the introgression of natural resistance genes via conventional breeding. In tomato and pepper, major viral threats such as tomato brown rugose fruit virus and TSWV have been managed using resistance genes including *Tm-22*, *Sw-5b*, and *Tsw*, which belong to the nucleotide-binding leucine-rich repeat protein family (De Ronde et al., 2013; Hak and Spiegelman, 2021). However, widespread deployment of these resistance genes imposes strong selection pressure on viral populations, driving the emergence of resistance-breaking strains (Nicaise, 2014; Luria et al., 2017; Gautam et al., 2023; Chinnaiah et al., 2023; Chinnaiah et al., 2026). Compounding this challenge, plant viruses exist as quasi-species, characterized by high genetic diversity and rapid mutation rates (10^-6^ to 10^-4^ substitutions per nucleotide), enabling rapid adaptation and temporal dominance of fitter variants (Sanjuán and Domingo-Calap, 2021; Zhang et al., 2015). This evolutionary plasticity complicates surveillance, resistance durability, and early detection of emerging variants, particularly under field conditions. Moreover, efficient transmission by insect vectors such as whiteflies and thrips accelerates viral spread across agroecosystems (Fiallo-Olivé et al., 2020; Whitfield et al., 2005). Together, these factors underscore the urgent need for rapid, sensitive, and field-deployable detection systems to enable early intervention and effective disease management. Originally described as a prokaryotic adaptive immune system, Clustered Regularly Interspaced Short Palindromic Repeats - CRISPR associated proteins (CRISPR-Cas) technology has since been adapted for genome editing, epigenome engineering, and gene therapy. The discovery of diverse Cas effector proteins has further expanded its application into pathogen detection (Makarova et al., 2013; Chandrasekaran et al., 2016; Wang et al., 2016; Borrelli et al., 2018; Li et al., 2019a; Yin et al., 2021; Bhat et al., 2022; Zhou et al., 2024; Kang et al., 2025). CRISPR-Cas systems are broadly classified into Class 1 (Types I, III, and IV) and Class 2 (Types II, V, and VI) (Mohanraju et al., 2016). The most widely used platforms—Cas9, Cas12a, and Cas13a—belong to Class 2 and correspond to Types II-A, V-A, and VI-A, respectively. Advances in Cas12a- and Cas13a-based platforms have enabled the development of sensitive and accurate systems for on-field detection of plant pathogens. These CRISPR-based diagnostic tools overcome many limitations of conventional methods, offering rapid, simple, and highly sensitive detection without the need for expensive equipment or controlled laboratory environments. Results can be visualized either by the naked eye or using low-cost, handheld devices such as battery-operated fluorometers.

This review examines the structural and functional features of key CRISPR–Cas systems, their application in engineering virus-resistant crops, and their emerging role in rapid, field-deployable detection of plant viruses in both hosts and vectors. Together, these advances position CRISPR technologies as integrated tools for resistance development, early detection, and effective disease management, with the potential to substantially reduce crop losses.

## 2 CRISPR Cas9 system: background and mechanism

CRISPR-Cas9 belongs to the Class 2 CRISPR-Cas system. CRISPR arrays consist of short, conserved repeat sequences (approximately 25–50 bp) interspersed with unique spacer sequences that originate from foreign genetic elements and guide target recognition in conjunction with Cas proteins (Semenova et al., 2011; Jinek et al., 2012). The CRISPR-Cas9 effector complex is composed of CRISPR RNA (crRNA), trans-activating crRNA (tracrRNA), and a multidomain CRISPR-associated Cas9 nuclease (Jinek et al., 2012, Jinek et al., 2014; Mohanraju et al., 2016).

**Figure 1:**
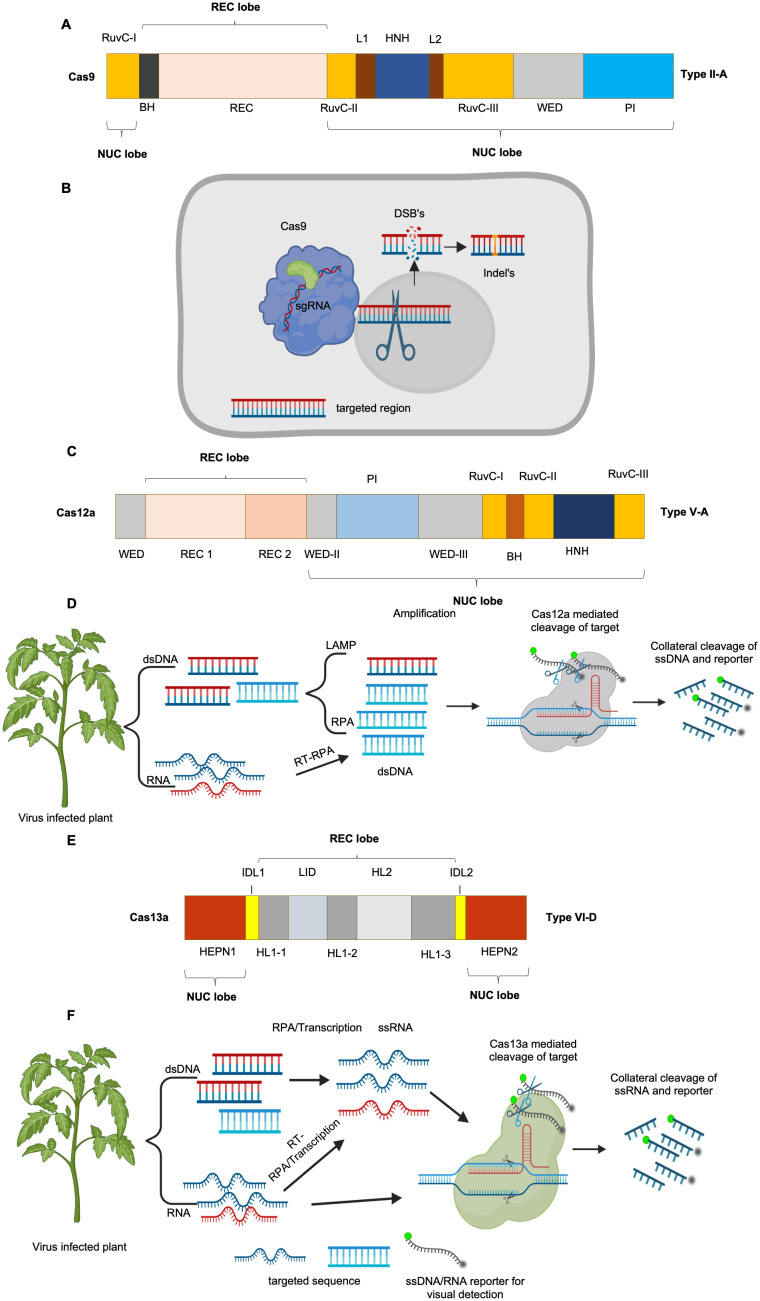
**(A)** Structural organization of the Cas9 protein, which adopts a bilobed architecture consisting of a recognition (REC) lobe and a nuclease (NUC) lobe. Major domains include the wedge domain (WED; grey), PAM-interacting domain (PI; light blue), recognition domain (REC; light orange), endonuclease domains RuvC (turquoise) and HNH (blue), and the bridge helix (BH; gold), connected by linker regions L1 and L2 (brown). **(B)** Schematic representation of Cas9-mediated induction of double-stranded DNA breaks (DSBs) at the target genomic locus. Repairing of DSBs by host DNA repair pathways results in insertions or deletions (indels), leading to disruption of gene function. **(C)** Structural organization of FnCas12a, which exhibits bilobed architecture like Cas9, with largely conserved domains and distinct linker regions (see image for comparison). **(D)** Schematic workflow for Cas12a-based detection of viral nucleic acids from infected plant samples. Target DNA or RNA is first enriched using amplification methods such as LAMP or RPA (for DNA) or RT-RPA (for RNA), followed by recognition and cleavage by the Cas12a–crRNA ribonucleoprotein (RNP) complex. Target recognition activates collateral cleavage of single-stranded DNA, including labeled reporter molecules, resulting in fluorophore release that can be visualized using a handheld fluorometer. **(E)** Structural organization of Cas13a, which, like Cas9 and Cas12a, adopts a bilobed architecture. Major domains include the HEPN nuclease domains (HEPN1 and HEPN2; red), intrinsically disordered linker regions (IDL1 and IDL2; yellow), and multiple helical domains (HL1–1 to HL1–3 and HL2; grey and light grey). **(F)** Schematic representation of Cas13a-mediated detection of viral nucleic acids from infected plant samples. Target DNA or RNA is amplified using RPA or RT-RPA and subsequently transcribed into RNA. The resulting RNA serves as a substrate for crRNA-guided Cas13a cleavage. Activation of collateral RNase activity leads to cleavage of single-stranded RNA reporters, releasing a fluorophore that can be detected using a handheld fluorometer.

Cas9 is an RNA-guided DNA endonuclease and a multidomain protein structurally organized into two major lobes: the recognition (REC) lobe and the nuclease (NUC) lobe (Tsai and Joung, 2016; Sung et al., 2018). The REC lobe comprises multiple helical domains (REC I–III), the bridge helix (BH), and associated regions involved in guide RNA binding and target recognition. The NUC lobe contains the catalytic domains RuvC (I–III) and HNH, along with the protospacer adjacent motif (PAM)-interacting domain, which together mediate site-specific DNA cleavage (Brouns et al., 2008; Jinek et al., 2012; Vazquez Reyes et al., 2017) (**Figure 1**).

The demonstration that CRISPR-Cas systems are transferable across bacterial species, coupled with the discovery that Cas9 activity can be guided by a single chimeric RNA combining crRNA and tracrRNA into a single-guide RNA (sgRNA), marked a turning point in the development of CRISPR-Cas as a versatile genome-editing tool (Sapranauskas et al., 2011; Jinek et al., 2012; Adli, 2018). The sgRNA directs Cas9 to a complementary target sequence, and upon base pairing, Cas9 induces a double-stranded DNA break (DSB) approximately 3–5 nucleotides upstream of a PAM sequence (typically NGG, where N represents any nucleotide) (Thyme et al., 2016; Mekler et al., 2017) (**Figure 1**).

DSBs generated by Cas9 are repaired by endogenous cellular DNA repair pathways, primarily non-homologous end joining (NHEJ) or homology-directed repair (HDR) (Lieber, 2010; Chang et al., 2017). Due to the error-prone nature of NHEJ, repair often results in small insertions or deletions (indels) that can introduce frameshift mutations or premature stop codons, thereby disrupting gene function. Targeted genome editing using *Streptococcus pyogenes* Cas9 (SpCas9) has since been widely applied across plant and animal systems to investigate gene function, elucidate disease mechanisms, improve agronomic traits, and develop resistance against pathogens.

Single-guide RNA (sgRNA) confers sequence specificity in CRISPR-mediated nucleic acid targeting, although its performance and mode of action vary across different Cas nuclease systems. Minimizing off-target effects remains a critical challenge, making rational sgRNA/crRNA design a key determinant of editing accuracy (Guo et al., 2021). Effective guide design requires consideration of sequence specificity, GC content, secondary structure, and potential off-target sites within the host genome. Several computational tools, including CRISPR-P 2.0, CRISPRdirect, CRISPR-GE, and CRISPR-PLANT, facilitate sgRNA selection by integrating genomic information and off-target prediction algorithms (Liu et al., 2017; Naito et al., 2015; Xie et al., 2017; Minkenberg et al., 2018). In cases where complete genome information is unavailable, off-target assessment using closely related species provides a practical alternative. Experimental validation of off-target effects can be performed using both cell-based and cell-free approaches; however, these methods often rely on whole-genome sequencing, increasing cost and limiting scalability (Liang et al., 2022; Cromer et al., 2023; Bao et al., 2021; Wienert et al., 2019; Crosetto et al., 2013; Tsai et al., 2015). To further enhance specificity, engineered Cas9 variants with reduced non-specific DNA interactions have been developed, including evoCas9, SpCas9-HF1, and eSpCas9 (Casini et al., 2018; Kleinstiver et al., 2016; Chen et al., 2018). Additional modifications, such as variants with relaxed protospacer adjacent motif (PAM) requirements, expand targetable genomic regions but may involve trade-offs in cleavage efficiency (Nishimasu et al., 2018). Collectively, these advances highlight the importance of integrating computational design, experimental validation, and enzyme engineering to achieve high-precision genome editing.

## 3 CRISPR-Cas9 applications in host plant resistance against viruses

The limited availability of broad-spectrum resistant cultivars, coupled with intensive reliance on chemical pesticides, has accelerated the emergence of more virulent viral strains capable of overcoming host resistance (Gautam et al., 2023; Chinnaiah et al., 2023). Most plant viruses rely on host susceptibility factors (also referred to as positive regulators) to complete their infection cycles. One of the most extensively exploited host factors is eukaryotic translation initiation factor 4E (eIF4E), which is required by many potyviruses for translation of their genomes (Singhal et al., 2026).

Targeted disruption of eIF4E genes using CRISPR-Cas9 has successfully conferred resistance or tolerance to multiple viruses across diverse crops. Representative examples include resistance to zucchini yellow mosaic virus, papaya ringspot virus-W, and cucumber vein yellowing virus in cucumber (Chandrasekaran et al., 2016); potato virus Y and pepper mottle virus in tomato (Kumar et al., 2022; Yoon et al., 2020); turnip mosaic virus in Chinese cabbage (Lee et al., 2023); tobacco etch virus in *Nicotiana benthamiana* (Kang et al., 2025); and Moroccan watermelon mosaic virus in watermelon (Pechar et al., 2022).

Beyond translation factors, CRISPR-Cas9–mediated knockout of TOBAMOVIRUS MULTIPLICATION 1 (TOM1), a positive regulator of tobamovirus replication, has been successfully used to confer resistance against ToBRFV and tomato mosaic virus in tomato (Kravchik et al., 2022; Sandhya et al., 2025), as well as tobacco mosaic virus in tobacco (Jogam et al., 2023). In addition, replication proteins of several begomoviruses interact with host factors such as ubiquitin-associated translation elongation factor (UbEF1B) and CCR4–NOT transcription complex subunit 3 (CCR4/NOT3) (Li et al., 2023). Targeted editing of UbEF1B and CCR4/NOT3 in *N. benthamiana* significantly attenuated disease symptoms and reduced viral loads of tomato yellow leaf curl China virus, tobacco curly shoot virus, tomato yellow leaf curl virus, and tomato leaf curl Yunnan virus (Li et al., 2023). For many viral pathogens, however, host susceptibility factors with minimal growth–defense trade-offs remain unidentified. In such cases, pathogen-targeted strategies, including expression of virus-specific sgRNAs via CRISPR-Cas9 systems, offer an alternative approach for inducing resistance.

The CRISPR–Cas9 system has been widely applied to target plant DNA viruses through both transgenic and transient expression approaches. Delivery of virus-specific sgRNAs in combination with Cas9 in model and crop systems such as *Nicotiana benthamiana*, *Arabidopsis thaliana*, and tomato has resulted in significant reductions in viral accumulation, including beet severe curly top virus, bean yellow dwarf virus, tomato yellow leaf curl virus, and cauliflower mosaic virus (Ji et al., 2015; Tashkandi et al., 2018; Liu et al., 2018). In addition, a reprogrammed *Francisella novicida* Cas9 (FnCas9) has been successfully employed to target RNA viruses, including cucumber mosaic virus and tobacco mosaic virus, demonstrating the versatility of CRISPR-based antiviral strategies (Zhang et al., 2018). However, the high mutation rates and quasi-species nature of viral populations enable rapid evolution and potential escape from CRISPR–Cas9-mediated targeting. To address this limitation, multiplexed strategies that simultaneously target multiple viral genomic regions and/or host susceptibility factors are likely to be essential for achieving durable and broad-spectrum resistance.

## 4 Nucleic acid detection using CRISPR-Cas systems

CRISPR-Cas12a- and Cas13a–mediated field-deployable detection platforms have emerged as powerful alternatives to conventional diagnostic tools. Advances in mechanistic understanding of CRISPR-Cas systems have enabled the development of Cas12a (formerly Cpf1; Type V-A) and Cas13a (formerly C2c2; Type VI-A)–based diagnostics that are increasingly applied to plant pathogen detection (Wang et al., 2020; Kaminski et al., 2021; Tian et al., 2022).

Both Cas12a and Cas13a belong to Class 2 CRISPR-Cas systems and are distinguished by their ability to exhibit collateral (trans) cleavage activity following target recognition—an activity not observed in Cas9 (Zetsche et al., 2015; Abudayyeh et al., 2017; Gootenberg et al., 2018; Chen et al., 2018) (**Figure 1**). Unlike Cas9, these systems do not require a tracrRNA. Structurally, both Cas12a and Cas13a are multidomain proteins containing recognition (REC) and nuclease (NUC) lobes.

Cas12a functions as an RNA-guided DNA nuclease, wherein CRISPR RNA (crRNA)-directed binding to double-stranded DNA triggers clof the target followed by collateral cleavage of single-stranded DNA (**Figure 1**). Target recognition requires a T-rich protospacer-adjacent motif (PAM). In contrast, Cas13a is an RNA-guided RNA endoribonuclease that recognizes single-stranded RNA targets and subsequently induces collateral cleavage of nearby ssRNA molecules (**Figure 1**). Exploiting this property with fluorescently labeled reporter probes enables rapid and sensitive visual detection. By designing virus-specific crRNAs and combining these systems with isothermal amplification methods, both DNA and RNA viruses can be detected with high accuracy.

CrRNA guides Cas12a and Cas13a nucleases to their target sequences, making guide design a critical determinant of detection specificity and robustness. Target regions should be selected from conserved viral sequences identified through alignment of diverse strains, while avoiding mutation-prone regions that may compromise assay sensitivity. Off-target complementarity within the host genome should also be minimized to reduce false-positive signals. For Cas13a-based detection, targeting the positive-sense RNA strand is particularly important in negative-sense or ambisense viruses, as this strand accumulates to higher levels during infection. Optimal crRNA design further requires minimal secondary structure and moderate GC content (approximately 40–60%) to ensure efficient target recognition and cleavage (Abudayyeh et al., 2016; Gootenberg et al., 2017). Cas12a- and Cas13a-based platforms offer rapid and sensitive detection, typically within 15–60 minutes, compared to conventional methods such as RT-PCR and ELISA, which may require 24–48 hours. Detection outputs can be visualized directly or using low-cost handheld fluorescence readers, eliminating the need for extensive downstream processing and reducing reliance on specialized laboratory equipment. In addition, both systems can detect viral nucleic acids directly from crude extracts, substantially reducing sample preparation time. However, multiplex detection remains a key challenge. Current approaches rely on fluorophore-labeled probes, requiring multiple distinct signals and more sophisticated instrumentation for discrimination, which may limit the development of fully field-deployable, instrument-free diagnostic platforms.

## 5 Cas12a-based plant virus detection

Foundational studies on CRISPR-Cas biology led to the development of highly sensitive nucleic acid detection platforms such as HOLMES and DETECTR (Zetsche et al., 2015; Chen et al., 2018; Li et al., 2018a, Li et al., 2018b; Gootenberg et al., 2018). DETECTR, developed by Chen et al. (2018), demonstrated that Cas12a–crRNA complexes cleave target DNA and activate collateral reporter cleavage, generating a fluorescent signal. Although initially applied to animal viruses such as human papillomavirus, these platforms have since been adapted for plant virus detection (**Table 1**).

**Table 1:** List of key plant viruses detected by Cas12a and 13a.

Targeted virus	Genome	Coupled methods	Probe	Visual detection	Reaction time	Reference	Starting template
Cas12a mediate detection of plant viruses							
MCMV	RNA	RT-RPA	fluorogenic ssDNA	Yes	45 min	Duan et al., 2022	RNA
PSTVd, TCDVd, TPMVd, CLVd, PCFVd, TASVd	RNA	RT-RPA	fluorogenic ssDNA	Yes	40 min.	Wang et al., 2025	RNA
TMV, PVX, PVY	RNA	RT- RPA	fluorogenic ssDNA	Yes	~30 min	Aman et al., 2020	RNA
TMV, TEV, PVX	RNA	RT-RPA, LFA	fluorogenic ssDNA	Yes	~2 hr	Marqués et al., 2022	RNA
AMV	RNA	RT-RPA, LFA	fluorogenic ssDNA, FAM and biotin	Yes	1 hr	Yan et al., 2025	RNA
ToMV, ToBRFV	RNA	RT-PCR	fluorogenic ssDNA	Yes	NM	Alon et al., 2021	RNA
Sorghum mosaic virus and rice stripe mosaic virus (RSMV)	RNA	RT-RPA	fluorogenic ssDNA	Yes	30 min	Wang et al., 2023	Crude extract
Sugarcane streak mosaic virus	RNA	RT-RPA	fluorogenic ssDNA	Yes	50 min	Gao et al., 2025	Crude extract
Citrus tristeza virus	RNA	RT-RPA	fluorogenic ssDNA	Yes	NM	Kim et al., 2024	RNA
Brassica Yellows Virus	RNA	RT-LAMP	fluorogenic ssDNA	Yes	1 hr	Xu et al., 2024	RNA
ChiLCV	DNA	RPA, LFA	DNase alert, FAM and biotin	Yes	~ 50 min	Paul et al., 2025	DNA
TSWV	RNA	RT-LAMP	fluorogenic ssDNA	yes	1hr	Shymanovich et al., 2024	Crude extract
BNYVV	RNA	RT-RPA	fluorogenic ssDNA	Not shown	1hr	Ramachandran et al., 2021	RNA
RSMV, Rice black-streaked dwarf virus	RNA	RT-LAMP	Yes	Yes	~1 hr	Zhu et al., 2022	RNA
Grapevine redblotch virus	RNA	RT-PCR	AuNP-based plasmonic readout	Yes	NM	Li et al., 2019b	RNA
Cas13a mediate detection of plant viruses							
TSWV	RNA	RPA-transcription	ssRNA fluorophore	NM	2:30 hrs	Zhang et al., 2021	RNA
CLRDV	RNA	Amplification free	RNaseAlert^®^ substrate	Yes	30 min.	Sheri et al., 2025	RNA, Crude extract
CMV	RNA	RT-Transcription	ssRNA fluorophore	Yes	2:30 hrs	Demissie et al., 2025	RNA, crude extract
TMV, TEV, PVX	RNA	Amplification free	ssRNA fluorophore	Yes	~30 min.	Marqués et al., 2022	Crude extract
ToBRFV, CGMMV, TuMV	RNA	Amplification free	RNaseAlert^®^ substrate	Yes	~30 min.	Hak et al., 2025	Crude extract

NM, Not mentioned in the study.

To improve sensitivity, Cas12a-based assays have been integrated with RT, RPA, and LAMP amplification methods (**Figure 1**), along with lateral flow and fluorescence-based readouts (Xu et al., 2023; Atçeken et al., 2022; Fu et al., 2025; Niu et al., 2025). Aman et al. (2020) developed a two-step RT-RPA–Cas12a assay for detection of tobacco mosaic virus, potato virus X, and potato virus Y, achieving sensitive detection using a portable fluorescence viewer. Similar LAMP–Cas12a platforms have been applied for detection of ssDNA plant viruses (Mahas et al., 2021).

Field-deployable RT-RPA–Cas12a assays have also been demonstrated using crude plant extracts, including detection of apple-infecting viruses (Jiao et al., 2021) and maize chlorotic mottle virus via lateral flow strips (Lei et al., 2023). The latter system achieved detection limits as low as 2.5 viral copies and enabled identification of virus in asymptomatic plant tissue. Collectively, Cas12a-based systems offer precise, sensitive, and visually interpretable detection of both DNA and RNA plant viruses.

## 6 Detection of plant viruses using Cas13a

Cas13a differs fundamentally from Cas12a in that it directly targets and cleaves single-stranded RNA. Target recognition depends on a non-G nucleotide within the 3′ protospacer flanking site (PFS). Given that approximately 70% of plant viruses possess RNA genomes, Cas13a represents a particularly attractive platform for virus detection (**Table 1**).

The SHERLOCK platform, initially developed by Gootenberg et al. (2017), combines RPA-mediated amplification with T7 transcription to generate RNA targets for Cas13a-mediated detection (**Figure 1**). This system enables detection down to the single-molecule level and can be adapted for both RNA and DNA targets. In plants, Marqués et al. (2022) demonstrated Cas12a- and Cas13a-based detection of tobacco mosaic virus, tobacco etch virus, and potato virus X, showing that Cas13a-based assays could detect viral RNA directly from crude plant extracts without prior amplification.

More recently, SHERLOCK-based Cas13a systems have been extended to viroid detection (Zhai et al., 2024). Hak et al. (2025) developed an extraction-free, 15-minute Cas13a assay for point-of-care detection, enabling visualization using a handheld fluorescence viewer or smartphone camera. This platform successfully detected ToBRFV, cucumber green mottle mosaic virus (CGMMV), and turnip mosaic virus (TuMV) at early stages of infection. The simplicity, speed, and minimal infrastructure requirements of Cas13a-based diagnostics suggest strong potential for rapid adoption in field and grower settings.

Commonly used LbCas12a and LbuCas13a exhibit comparable catalytic efficiencies (10^5^;–10^6^ M^-1^ s^-1^), which constrain amplification-free detection sensitivity to the picomolar range. In these systems, signal generation through collateral reporter cleavage is strongly dependent on target nucleic acid concentration (Huyke et al., 2022). To enhance sensitivity, Cas12a-based assays are typically coupled with nucleic acid amplification methods such as RT-PCR, RPA, or LAMP, although these steps increase overall assay time and complexity. In contrast, Cas13a, as an RNA-guided RNA nuclease, is not directly compatible with these DNA-based amplification workflows. Improving the limit of detection for Cas13a-based platforms therefore relies on enhancing intrinsic catalytic efficiency (kcat/Km), which may be achieved through engineering of the HEPN RNase domains.

## 7 Conclusions and future prospects

Plant viruses pose a growing and persistent threat to global crop production, necessitating solutions that can both limit pathogen spread and enable early, scalable detection. This review synthesizes recent advances in CRISPR-based approaches across two complementary fronts: host resistance engineering using Cas9 and pathogen detection using Cas12a and Cas13a platforms. Together, these studies highlight how CRISPR technologies are transitioning from experimental systems to practical tools for plant disease management and surveillance.

On the resistance front, CRISPR-Cas9–mediated editing of host susceptibility factors has emerged as a durable strategy for virus control. Among the most extensively studied targets are eukaryotic initiation factors, particularly eIF4E and its isoforms, which have been edited to confer resistance against multiple potyviruses across diverse crops. In addition, disruption of host genes required for viral replication, including TOM1, UbEF1B, and CCR4/NOT3—has shown promise against a broader range of pathogens. These host-directed approaches offer a key advantage by reducing the likelihood of resistance breakdown driven by rapid viral evolution. CRISPR-edited crops lacking foreign DNA insertions are approved for commercialization in several countries, including the United States. In contrast, similar crops in the European Union are regulated under conventional GMO frameworks, requiring more stringent approval processes. This divergence in regulatory approaches—driven in part by concerns over potential off-target effects and differing policy interpretations—poses a significant barrier to the global commercialization and adoption of CRISPR-edited crops (Ahmad et al., 2024).

In parallel, CRISPR-based diagnostic platforms have demonstrated substantial potential for rapid and sensitive plant virus detection. Cas12a-based assays are currently the most widely explored, particularly for RNA viruses, and have been successfully integrated with amplification methods such as RT-PCR, RPA, LAMP, and lateral flow assays. Many of these systems achieve detection within 30–60 minutes using fluorescence or visual readouts, underscoring their suitability for time-sensitive diagnostics and large-scale deployment.

By contrast, Cas13a-based detection remains comparatively underdeveloped, having been evaluated for only a limited number of RNA viruses, including TSWV, CLRDV, and CMV. While Cas13a offers direct RNA targeting and high specificity, current fluorescence-based workflows typically require 2–2.5 hours to complete. Reducing assay times to under 30 minutes remains a critical technical challenge for improving its practicality, particularly for high-throughput and field-oriented applications.

The contrasting performance of Cas12a and Cas13a platforms reflects fundamental mechanistic differences. Cas12a targets double-stranded DNA and triggers collateral cleavage of single-stranded DNA, enabling rapid signal amplification following reverse transcription and pre-amplification. In contrast, Cas13a directly targets RNA and induces collateral cleavage of single-stranded RNA, offering a conceptually streamlined approach for RNA virus detection that is currently constrained by slower kinetics and less mature assay designs. Continued optimization informed by these mechanistic distinctions will be essential for improving assay speed, sensitivity, and robustness, as well as for enabling multiplexed detection and strain-level resolution, which are increasingly important given the complexity of plant disease outbreaks.

Despite strong experimental progress, both Cas12a and Cas13a platforms remain largely pre-commercial. Ongoing efforts focus on shortening detection times, reducing costs, enhancing assay stability, and simplifying workflows. Future research must prioritize translation into scalable, end-user–oriented products, including portable devices and low-infrastructure kits capable of operating outside centralized laboratories and contributing directly to broader surveillance efforts. To enhance sensitivity and cost-effectiveness, future research should also increasingly focus on enzyme engineering, CRISPR-based electrochemical biosensors, microfluidic diagnostic platforms, and AI-assisted pathogen detection.

For CRISPR-based point-of-care diagnostic platforms, reagent stability remains a major challenge, particularly the activity of Cas proteins and the integrity of guide RNAs under fluctuating temperature conditions. Factors such as storage temperature, buffer composition, protein concentration, and repeated freeze–thaw cycles can significantly affect enzyme performance and complex stability (Wöll et al., 2020; Kumar et al., 2019; Hauptmann et al., 2018; Garidel et al., 2015). Approaches such as lyophilization and the use of stabilizing agents (e.g., 5–10% trehalose) have shown promise in improving field compatibility; however, current strategies remain insufficient for ensuring long-term stability under ambient conditions (Saini et al., 2026; Lagner et al., 2025). Future efforts should therefore focus on enhancing formulation stability to reduce reliance on cold-chain logistics while maintaining enzymatic activity. In addition, the high cost of Cas enzymes and guide RNAs represents a significant barrier to large-scale commercialization. Addressing this challenge will require both cost-reduction strategies and the development of engineered Cas variants with improved stability and activity across a broader range of environmental conditions. Recent advances, such as the development of thermostable Cas12b variants (e.g., eBrCas12b, with enhanced thermal stability from 62 °C to 67 °C), highlight the potential of protein engineering to improve assay robustness and compatibility with isothermal amplification systems (Nguyen et al., 2023).

If successfully deployed, CRISPR-based diagnostics could overcome key limitations of existing nucleic acid– and protein-based detection methods, which are often expensive, time-consuming, and technically demanding. In contrast, CRISPR-enabled systems provide a pathway toward rapid, affordable, and decentralized surveillance. Their ultimate impact will depend on integration into coordinated epidemiological frameworks, where early detection can inform real-time decision-making, regional risk assessment, and outbreak containment.

In summary, integrating CRISPR-mediated resistance strategies with next-generation CRISPR diagnostics offers a unified and forward-looking framework for managing plant pandemics. Developing efficient, scalable detection and surveillance tools represents a critical first step toward mitigating disease spread and reducing economic losses. Extending these approaches beyond plant tissues to include vector-based surveillance presents an additional opportunity for earlier intervention and improved disease forecasting as CRISPR technologies continue to mature.
